# Antihypertensive and Antihypertrophic Effects of Acupuncture at PC6 Acupoints in Spontaneously Hypertensive Rats and the Underlying Mechanisms

**DOI:** 10.1155/2017/9708094

**Published:** 2017-02-15

**Authors:** Juan-Juan Xin, Jun-Hong Gao, Yuan-Yuan Wang, Feng-Yan Lu, Yu-Xue Zhao, Xiang-Hong Jing, Xiao-Chun Yu

**Affiliations:** Institute of Acupuncture and Moxibustion, China Academy of Chinese Medical Sciences, 16 Nanxiaojie, Dongzhimennei, Beijing 100700, China

## Abstract

The aim of this work is to investigate the effect of electroacupuncture (EA) at PC6 on the hypertension and myocardial hypertrophy in spontaneously hypertensive rats (SHRs). Thirty SHRs were randomized into model, SHR + EA, and SHR + Sham EA group with WKY rats as normal control. EA was applied once a day in 8 consecutive weeks. The blood pressure (BP), cardiac function, and hypertrophy as well as the underlying mechanisms were investigated. After EA treatment, the enhanced BP in SHR + EA group was significantly lower compared to both the period before EA and model group. Echocardiographic, morphological studies showed that the enhanced left ventricular anterior and posterior wall end-diastolic (LVAWd and LVPWd) thickness, diameters and cross-sectional area (CSA) of cardiac myocyte, as well as the ratio of heart weight to body weight (HW/BW), were markedly diminished in SHR + EA group, while the reduced left ventricular ejection fraction, left ventricular short axis fraction shortening, and E/A ratio were significantly ameliorated. The levels of Angiotensin-converting enzyme (ACE) and Angiotensin II Type 1 and 2 receptors (AT1R, AT2R) in SHRs were also significantly attenuated by EA. The results suggest that EA at bilateral PC6 could arrest the hypertension development and ameliorate the cardiac hypertrophy and malfunction in SHRs, which might be mediated by the regulation of ACE, AT1R, and AT2R.

## 1. Introduction

Hypertension is a key risk factor for various cardiovascular disorders, which affects approximately 20–50% adult populations in developed countries [1, 2]. With the cardiac insult of persistently increased blood pressure, an adaptive response will be evoked that eventually leads to the formation of left ventricular hypertrophy (LVH), which, in turn, increases the occurrence of cardiac arrhythmias, myocardial infarction, heart failure, and even sudden cardiac death [3, 4].

Increasing evidence has suggested that both the overactive sympathetic nervous system and endocrine factors paly crucial roles in the pathological progression of persistent hypertension, in which the function of renin-angiotensin system (RAS) has been highly emphasized due to its impact on the circulating volume and electrolyte balance, as well as the haemodynamic stability [5]. To date, much progress has been made in the research on the RAS cascade, and the Angiotensin-converting enzyme (ACE) inhibitors and Angiotensin II (Ang II) type 1 receptor (AT1R) blockers are deemed as the “gold standard” for antihypertensive therapies [6].

Although novel antihypertensive pharmacological therapies have been developed nowadays, only 5–30% of patients with hypertension achieve adequate blood pressure control, which is partially caused by low compliance to the combination of multiple antihypertensive medicines and their side effects [7]. Notably, in recent years the clinical trials have revealed that acupuncture is effective in lowering increased blood pressure [8, 9]. Previous experimental studies also showed that acupuncture at Taichong (Lv3) [10], Baihui (GV20) [11], and Zusanli (ST36) [12] could attenuate the increased blood pressure in SHR. However, the effectiveness achieved in the studies was not so satisfactory based on the fact that during and after acupuncture treatment the blood pressure was continuously enhancing in the acupuncture treating group. The accumulated experimental and clinical studies showed that PC6 is the most commonly used and more effective acupoint in the treatment of the cardiovascular diseases [8, 13]. Thus, in the present study we select the bilateral PC6 to be stimulated by acupuncture so as to explore the antihypertensive and antihypertrophic effects of acupuncture and the underlying mechanisms. In addition, the contribution of ACE to the effects of acupuncture on the functional and structural pathological progression of persistent hypertension has not been addressed yet. By using the spontaneously hypertensive rats (SHRs), the possible roles of ACE and Ang II receptors (AT1R, AT2R) in the mediation of the inhibitory effects of EA (8 w) on hypertension and myocardial hypertrophy were also investigated in our present study.

## 2. Methods

### 2.1. Animal Preparation

Thirty male SHRs at the age of 12 weeks and 10 male Wistar-Kyoto (WKY) rats of the same age, weighing 240–270 g, were obtained from Vital River Laboratories (Certificate number SCXK 2012-0001, Beijing, China). The rats were housed in cages at 24 ± 1°C and humidity of 50 ± 5% under a 12-hour light/dark cycle and received standard diet and water ad libitum. The experiments were conducted in accordance with the Guide for Use and Care of Medical Laboratory Animals from Ministry of Public Health of China.

### 2.2. Animal Grouping and Electroacupuncture Treatment

The rats were randomly divided into 4 groups: WKY group (*n* = 10), SHR group (*n* = 10), SHR + EA group (*n* = 10), and SHR + Sham group (*n* = 10). Under isoflurane inhalation anesthesia, the animals in SHR + EA group were subjected to electroacupuncture treatment at bilateral Neiguan acupoints (PC6) which were located at a point 1.5 cm proximal to the palm crease just above the median nerve. Sterilized disposable stainless steel needles (0.3 mm × 15 mm, Global Brand, Suzhou, China) were penetrated 2 mm into the subcutis beneath the acupoints and connected with a Han's Acupoint and Nerve Stimulator (Model HANS-200A, Ji Sheng Medical Technology Co., Ltd., Nanjing, China). Electrical stimulation (2/15 Hz, 1 mA) proceeded for 30 minutes per day, for a period of 8 weeks [14]. In SHR + Sham group, needles were inserted in the superficial layer of PC6 with no electrical stimulation applied, to expel the interference caused by needle insertion, grabbing, and anesthesia on EA's effects.

### 2.3. Blood Pressure Measurement

Under conscious condition, blood pressure levels were recorded by using a CODA Mouse & Rat Tail-Cuff Blood Pressure System (Kent Scientific Co., Connecticut, USA), including the systolic blood pressure (SBP), diastolic blood pressure (DBP), and the mean arterial blood pressure (MAP), as described in previous study [15]. The measurement was conducted once every week at 9–11 am in a quiet room and the blood pressure of each rat was tested for three consecutive times to calculate the mean value.

### 2.4. Echocardiographic Analysis

The structural and functional changes among the four groups were tested by Vevo 770 High Resolution Imaging Systems (Visual Sonics, Toronto, Canada) with a 17.5 MHz linear array transducer (model 716) once every two weeks. All rats were anesthetized with 1.5–2.0% isoflurane. Two-dimensional cine loops and guided M-mode frames were recorded from the parasternal short and long axis to assess the left ventricular anterior wall end-diastolic (LVAWd) thickness, left ventricular end-diastolic internal diameter (LVIDd), left ventricular posterior wall end-diastolic (LVPWd) thickness, left ventricular ejection fraction (LVEF), and left ventricular short axis fraction shortening (LVFS) [16]. Pulsed-wave Doppler early to late transmitral peak diastolic flow velocity (E/A) ratio was measured to assess diastolic function as described previously [17]. All measured and calculated indices were presented as the average of three consecutive cardiac cycles.

### 2.5. Histological Measurement

Six rats in each group were killed after 8 weeks of EA treatment, and the hearts were removed and washed with 4°C saline. Both body weight (BW) and heart weight (HW) were determined, and the HW/BW ratio was calculated to evaluate the hypertrophic response to overload blood pressure. The other 3 rats in each group were anaesthetized by 10% urethane and transcardially perfused with 250 mL of 0.9% saline immediately followed by 300 mL of 4% paraformaldehyde in 0.1 M phosphate buffered solution (PB, pH 7.4). The left ventricular (LV) section was cut off transversely at the midventricular level for paraffin sectioning [18]. The myocardial sections (5 *μ*m) were deparaffinized and rehydrated and stained with hematoxylin and eosin. The images were captured by a digital camera connected to a microscope (Pannoramic MIDI/250, 3D HISTECH, Hungary). Diameters and cross-sectional area of cardiac myocyte were measured to represent the development of hypertrophy [19].

### 2.6. Immunoradiometric Assay of ACE in the Serum and Heart Tissue

Concentrations of ACE in heart tissue and serum were measured by commercial radioimmunoassay kits (Beijing Sino-UK Institute of Biological Technology Company, China) following the company's protocol.

### 2.7. Western Blotting

Heart tissues were lysed in RIPA buffer containing phosphatase and protease inhibitors (Roche Complete, Roche Diagnostics, Mannheim, Germany). The protein concentration in the supernatant was determined using the BCA method with a bovine serum albumin standard. Equal amount of total protein was subjected to SDS-PAGE and blotted on NC membrane (Millipore, Billerica, MA, USA). The blots were blocked with 5% defatted milk powder in Tris-buffered saline (TBS) buffer and then incubated with the respective primary antibodies (Mouse Anti-Angiotensin II Type 1 Receptor 1 : 500, Abcam, UK; Rabbit Anti-Angiotensin II Type 2 Receptor 1 : 1000, Abcam, UK; Mouse Anti-GAPDH 1 : 20000, TDY Biotech Co., Ltd., Beijing, China) for overnight at 4°C. The membrane was washed with TBS and incubated with horse radish peroxidase-conjugated goat anti-mouse or rabbit IgG (1 : 10000; TDY Biotech Co., Ltd., Beijing, China) for 40 min at room temperature. The targeted proteins were detected by using enhanced chemiluminescence system (Millipore, Billerica, MA, USA). The quantification of band intensity was carried out using Image-Pro Plus software. Band densities were normalized to individual GAPDH internal control.

### 2.8. Statistical Analysis

All data were expressed as mean ± standard deviation (SD). Statistical analysis was performed using one-way analysis of variance (ANOVA) followed by Turkey post hoc test or repeated measures ANOVA with Bonferroni post hoc test was used for multiple comparisons. Changes of blood pressure in the same group were compared statistically by a paired *t*-test. A probability of less than 0.05 was considered to be statistically significant.

## 3. Results

### 3.1. EA Treatment Attenuates the Blood Pressure Elevation in SHR Rats

**Figure 1:**
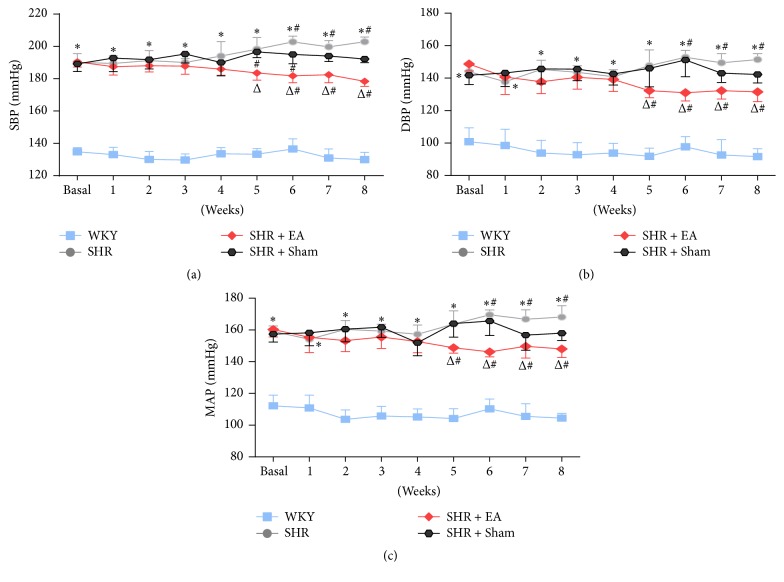
The antihypertensive effects of EA on the systolic (a), diastolic (b), and mean arterial pressure (c) in WKY and SHR. Blood pressure was measured by tail-cuff method before and after 1, 2, 3, 4, 5, 6, 7, and 8 weeks of EA treatment. ^#^*P* < 0.05 versus basal level; ^*∗*^*P* < 0.05 versus WKY; ^Δ^*P* < 0.05 versus SHR (*n* = 6 each group).

Noninvasive blood pressure recording showed that the blood pressure of SHR significantly elevated and remained on an increasing trend as compared with the WKY controls (*P* < 0.05). Following 5 weeks of EA treatment, the systolic blood pressure (SBP) of SHR + EA rats was slightly decreased and maintained stable from the 5th to 8th week, which was markedly lower than that of SHR (*P* < 0.05, Figure 1(a)). In addition, changes of diastolic and mean blood pressure followed the same pattern as the SBP (Figures 1(b) and 1(c)), indicating that 8-week EA at PC6 was effective in lowering all phases of blood pressure in SHR rats. Overall, as compared with basal levels, all three types of blood pressure were moderately increased in SHR (*P* < 0.05) while slightly depressed in SHR + EA rats (*P* < 0.05) from the 5th week.

### 3.2. Ameliorating Effects of EA on Myocardial Hypertrophy in SHR

#### 3.2.1. Echocardiographic Parameters

**Figure 2:**
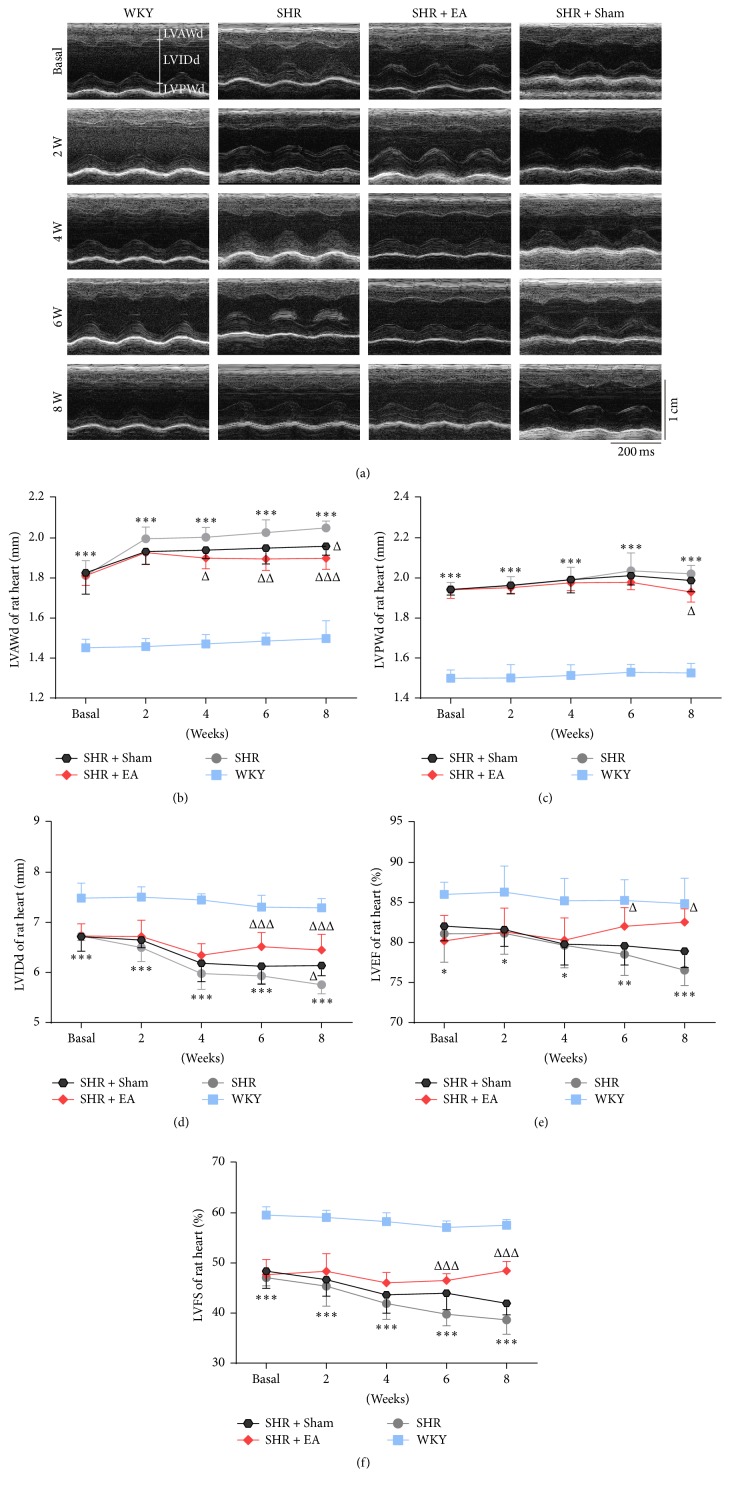
EA restored the structural and functional impairment of left ventricle in SHR. (a) Representative echocardiograms of rat hearts before and after 2, 4, 6, and 8 weeks of EA treatment for determination of the ventricle wall thickness. (b), (c), (d), (e), and (f) showed the improvement of myocardial hypertrophy and malfunction in SHR, in terms of the LVAWd, LVPWd, LVIDd, LVEF, and LVFS, respectively. ^*∗*^*P* < 0.05, ^*∗∗*^*P* < 0.01, and ^*∗∗∗*^*P* < 0.001 versus WKY; ^Δ^*P* < 0.05, ^ΔΔ^*P* < 0.05, and ^ΔΔΔ^*P* < 0.05 versus SHR (*n* = 6 each group).

**Figure 3:**
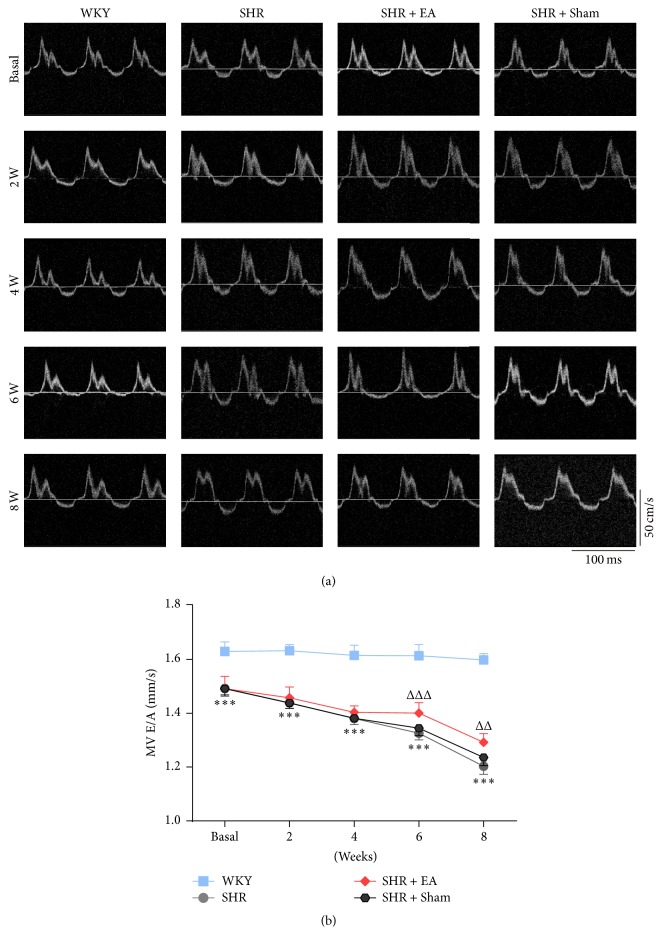
EA improved the pulsed-wave Doppler early to late transmitral peak diastolic flow velocity (E/A) ratio in SHR. (a) Representative pulse-wave Doppler echocardiograms of mitral inflow before and after 2, 4, 6, and 8 weeks of EA treatment. (b) E/A ratio was decreased in SHR when compared with that in WKY, which was reversed with EA treatment. ^*∗∗∗*^*P* < 0.001 versus WKY; ^ΔΔ^*P* < 0.05 and ^ΔΔΔ^*P* < 0.05 versus SHR (*n* = 6 each group).

By using echocardiographic analysis, the structural and functional alterations of left ventricle in SHR rats were observed, including LVAWd, LVPWd, LVIDd, LVEF, LVFS, and E/A ratio. As shown in Figure 2(a), the definitions of LVAWd, LVPWd, and LVIDd have been labeled segmentally. Compared with WKY controls, both the left ventricular anterior (LVAWd) and posterior wall end-diastolic (LVPWd) thickness were significantly increased in SHR throughout the course of the study (*P* < 0.001, Figures 2(b) and 2(c)). However, the anterior and posterior wall thickness in SHR was reduced markedly after 4- and 8-week EA treatment, respectively (*P* < 0.05). Associated with the increased wall thickness, the LVIDd in SHR rats was lower than that of WKY controls (*P* < 0.001, Figure 2(d)), which was improved after 6-week EA treatment (*P* < 0.001). LVEF, LVFS, and E/A ratio represent the functional indexes of myocardial systolic and diastolic capacity of left ventricle. During 8 weeks' observation, the LVEF and LVFS were continuously decreased in SHR as compared with those of WKY controls (*P* < 0.05, *P* < 0.01, *P* < 0.001), however, both of which have been significantly enhanced after 6-week EA treatment (Figures 2(e) and 2(f), *P* < 0.05, *P* < 0.001). In addition, the pulsed-wave Doppler results showed that the E/A ratio was dramatically declined in SHR rats (*P* < 0.001) while markedly restored in SHR + EA group (Figure 3, *P* < 0.01, *P* < 0.001). These data suggested the development of myocardial structural and functional impairment in SHR from ages 12 to 20 weeks, while repeated EA treatment is effective in attenuating the myocardial hypertrophy and cardiac malfunction.

#### 3.2.2. Histological Measurement and Organ Weights

**Figure 4:**
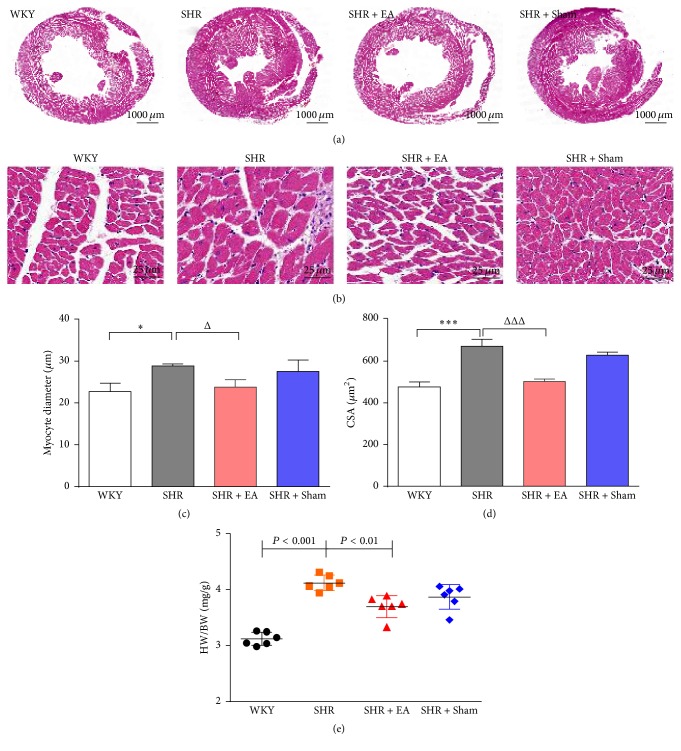
Effect of EA treatment on histology of myocardial tissue. The tissue was taken after 8 weeks of EA treatment from SHR or at the same time course from WKY and stained by hematoxylin and eosin. (a) H&E stained cross-sections of rat hearts in WKY, SHR, SHR + EA, and SHR + Sham group (bar = 1000 *μ*m). (b) Representative histology of myocardial tissue at higher magnification, indicating obvious myocyte hypertrophy, vacuolar degeneration, and inflammation cells infiltration in SHR, which were ameliorated by EA treatment (bar = 25 *μ*m). (c) Quantitative analysis of myocyte diameter of the cross-sectional tissue slices of heart. ^*∗*^*P* < 0.05 versus WKY; ^Δ^*P* < 0.001 versus SHR (*n* = 3 each group). (d) Quantitative analysis of cross-sectional area (CSA) of the cross-sectional tissue slices of heart. ^*∗∗∗*^*P* < 0.001 versus WKY; ^ΔΔΔ^*P* < 0.001 versus SHR (*n* = 3 each group). (e) The heart weight/body weight ratio (HW/BW ratio, mg/g) was estimated after 8 weeks of EA treatment (*n* = 6 each group).

The histological alteration in myocardial tissue was assessed by hematoxylin and eosin staining. As shown in Figures 4(a) and 4(b), myocyte hypertrophy, vacuolar degeneration, and inflammation cells infiltration were found in the myocardial tissue of SHR. Meanwhile, compared with WKY, the myocyte diameter and cross-sectional area of the cross-sectional tissue were significantly enlarged in SHR (Figures 4(c) and 4(d), *P* < 0.05), which could be attenuated by EA treatment (*P* < 0.05). On the cessation of 8-week EA treatment, the heart was removed and heart weight (HW)/body weight (BW) ratio was measured to assess the overall increased heart mass resulting from myocardial hypertrophy (Figure 4(e)). The HW/BW ratio in SHR rats was significantly higher than that of WKY controls (*P* < 0.001), whereas SHR + EA normalized the ratio versus SHR rats (*P* < 0.01). Additionally, the HW/BW ratio in SHR + Sham rats was moderately decreased, but with no statistical difference as compared with SHR rats.

#### 3.2.3. Involvement of ACE, AT1R, and AT2R in the EA-Induced Antihypertensive and Antihypertrophic Effects

**Figure 5:**
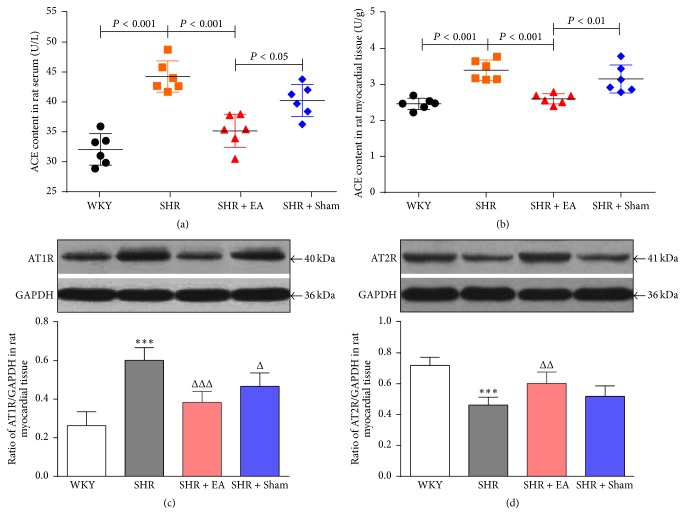
The roles of ACE, AT1R, and AT2R in the cardiac protective effects of EA on SHR. The concentration of ACE in serum (a) and heart tissue (b) measured by immunoradiometric assay. Western blotting analysis of AT1R (c) and AT2R (d) protein in left ventricular myocardium after 8 weeks of EA treatment. ^*∗∗∗*^*P* < 0.001 versus WKY; ^Δ^*P* < 0.05, ^ΔΔ^*P* < 0.01, and ^ΔΔΔ^*P* < 0.001 versus SHR (*n* = 6 each group).

Immunoradiometric assay showed that the levels of ACE in SHR were significantly higher than WKY, in both serum and heart tissue (Figures 5(a) and 5(b), *P* < 0.001). In addition, western blotting revealed that local expression of AT1R in SHR was significantly higher compared to WKY (Figure 5(c), *P* < 0.001), whereas AT2R was markedly decreased (Figure 5(d), *P* < 0.001). Notably, EA was effective in reversing the expression patterns of ACE and AT1R and AT2R in SHR (*P* < 0.01, *P* < 0.001), which might mediate the inhibitory effects of EA on myocardial hypertension and hypertrophy.

## 4. Discussion

Since the hypertension in most of the patients primarily or genetically caused enhancement of blood pressure, we selected SHR, a typical genetic hypertension animal model, to investigate the antihypertensive and antihypertrophic effects and the underlying mechanisms in the present study. Although previous studies showed a significant inhibition of hypertension was achieved by acupuncture in SHRs, the antihypertensive effect of acupuncture is actually limited more or less, which may be caused by the fact that the acupoints being stimulated in their studies are not optimal ones.

In the present study, we investigated the antihypertensive and antihypertrophic effects of EA at PC6 on SHR and the underlying mechanisms. Our data showed that, as compared with WKY controls, the systolic, diastolic, and mean arterial pressure were significantly elevated in SHR, which results in obvious structural and functional impairments in the myocardial tissue, including increased myocyte diameter, cross-sectional area, and anterior and posterior wall thickness, as well as reduced LVFS and E/A ratio. However, the increased blood pressure and hypertrophy and malfunction of myocardium were diminished by 8-week EA treatment. More interestingly, during the period from 4th to 8th weeks of EA treatment the increased blood pressure of the rats in SHR + EA group was significantly reduced, not only as compared with model group, but also even as compared with the rats themselves in SHR + EA group before the EA treatment, which means that the course of the hypertension development was arrested. Obviously, acupuncture applied at PC6 acupoints produced stronger antihypertensive effect than the aforementioned experimental studies in which the acupoints including Taichong (LV3), Baihui (GV20), and Zusanli (ST36) were selected only referring to the theory of Traditional Chinese Medicine without consideration of the anatomic or neurological aspects.

Morphological studies revealed that in the dorsal roots or dorsal horns there are dichotomizing and/or convergent neurons which are distributed to both somatic tissues and corresponding visceral organs [20, 21]. Anatomically, heart is known to be innervated by lower cervical and upper thoracic segments, both of which also innervate the somatic area around PC6 acupoint. In addition, it is well known that median nerves pass through the tissues beneath PC6 acupoint, while stimulation of median nerves was shown to lower the enhanced blood pressure [22]. The abovementioned facts provide the anatomical basis or segmental mechanisms for acupuncture at PC6 to regulate the cardiac pumping function and blood pressure. Actually, there is also accumulated clinical and experimental evidence showing that PC6 was more frequently used and more effective acupoint in the treatment of cardiovascular disorders including hypertension [23–25]. Consistent with the supporting background above, results of the present study suggest that a stronger antihypertensive effect could be produced by EA applied at PC6 acupoints.

Clinical studies have suggested that pharmacological inhibition on RAS is a critical component in the treatment of hypertension, in which the ACE inhibitors and Angiotensin 2 receptor blockers are the effective and most commonly used agents [26, 27]. However, the achievements in blood pressure control and myocardioprotection in clinic are still suboptimal due to low medicine compliance and multiple side effects, such as cough and angioedema [28].

Currently, increasing evidence demonstrated the promising effectiveness of acupuncture treatment on blood pressure control in both humans and animals [29, 30]. It has been shown that EA could attenuate the elevated blood pressure and be of benefit in restoring cardiac hypertrophy in SHR by enhancing NO/NOS activity and reducing the level of IL-6, STAT3, BNP, and so on [31–33]. Additionally, the EA-induced suppression on the pathological progression from hypertension to cardiovascular remodeling in SHR was also likely mediated via the downregulation of Ang 2, ATIR, endothelin type A receptor (ETAR), and endothelin-1 (ET-1) [34, 35]. The attenuation of apelin expression and function in rostral ventrolateral medulla (RVLM) neurons, which represents the major source of excitatory output to sympathetic preganglionic neurons, might be a central pathway involved in antihypertensive effects of EA on stress-induced hypertension [36]. However, there is no evidence showing whether or not ACE and AT2R are involved in mediating the EA-induced antihypertensive and antihypertrophic effects yet. With immunoradiometric and western blotting assay, we found that in SHR + EA group the enhanced ACE and AT1R were attenuated, while the reduced AT2R was elevated, respectively, after 8-week EA treatment. However, Sham EA can hardly lower the increased blood pressure in SHR rats and also has no obvious impact on the RAS. Our results suggest that the antihypertensive and antihypertrophic effects of EA at PC6 on SHR rats might be mediated by its modulation on the elements of RAS, including ACE, AT1R, and AT2R.

## 5. Conclusions

In conclusion, the results of the present study suggests that 8-week EA at PC6 attenuates significantly the increased blood pressure and the myocardial hypertrophy in SHR, which might be mediated by downregulation of enhanced both ACE and AT1R, as well as upregulation of the diminished expression of AT2R.
